# Phase I clinical trial of intra‐bone marrow cotransplantation of mesenchymal stem cells in cord blood transplantation

**DOI:** 10.1002/sctm.20-0381

**Published:** 2020-12-14

**Authors:** Tatsunori Goto, Makoto Murata, Tetsuya Nishida, Seitaro Terakura, Sonoko Kamoshita, Yuichi Ishikawa, Yoko Ushijima, Yoshiya Adachi, Satoshi Suzuki, Katsuyoshi Kato, Akihiro Hirakawa, Satoshi Nishiwaki, Nobuhiro Nishio, Yoshiyuki Takahashi, Yoshihisa Kodera, Tadashi Matsushita, Hitoshi Kiyoi

**Affiliations:** ^1^ Department of Hematology and Oncology Nagoya University Graduate School of Medicine Nagoya Japan; ^2^ Center for Advanced Medicine and Clinical Research Nagoya University Hospital Nagoya Japan; ^3^ Department of Pediatrics Nagoya University Graduate School of Medicine Nagoya Japan; ^4^ Department of Promotion for Blood and Marrow Transplantation Aichi Medical University School of Medicine Nagakute Japan; ^5^ Department of Transfusion Medicine Nagoya University Hospital Nagoya Japan

**Keywords:** cord blood transplantation, engraftment, graft‐vs‐host disease, intra‐bone marrow, mesenchymal stem cell

## Abstract

Mesenchymal stem cells (MSCs) have immunomodulatory properties and support hematopoiesis in the bone marrow (BM). To develop a new strategy to not only prevent graft‐vs‐host disease (GVHD) but also to enhance engraftment, a phase I trial of cord blood transplantation (CBT) combined with intra‐BM injection of MSCs (MSC‐CBT) was designed. Third‐party BM‐derived MSCs were injected intra‐BM on the day of CBT. The conditioning regimen varied according to patient characteristics. GVHD prophylaxis was tacrolimus and methotrexate. The primary endpoint was toxicity related to intra‐BM injection of MSCs. Clinical outcomes were compared with those of six controls who received CBT alone. Five adult patients received MSC‐CBT, and no adverse events related to intra‐BM injection of MSCs were observed. All patients achieved neutrophil, reticulocyte, and platelet recoveries, with median times to recoveries of 21, 35, and 38 days, respectively, comparable with controls. Grade II‐IV acute GVHD developed in three controls but not in MSC‐CBT patients. No patients developed chronic GVHD in both groups. At 1 year after transplantation, all MSC‐CBT patients survived without relapse. This study shows the safety of MSC‐CBT, and the findings also suggest that cotransplantation of MSCs may prevent GVHD with no inhibition of engraftment. This trial was registered at the University Hospital Medical Information Network Clinical Trials Registry as number 000024291.

## Graphical abstract

Mesenchymal stem cells (MSCs), which were derived from bone marrow (BM) of third‐party donor, were injected intra‐BM of recipient 4 hours before cord blood transplantation (CBT). This study shows the safety of CBT combined with intra‐BM injection of MSCs, and also suggests that co‐transplantation of MSCs may prevent graft‐vs‐host disease without inhibition of engraftment.

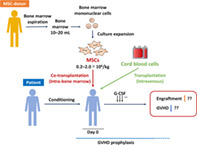

**Lessons learned**

- Mesenchymal stem cells (MSCs) support hematopoiesis in the bone marrow and have immunomodulatory properties.
- MSCs have potencies to enhance engraftment and to prevent graft‐versus‐host disease (GVHD) after allogeneic hematopoietic cell transplantation.
- This study shows the safety of intra‐bone marrow co‐transplantation of MSCs in cord blood transplantation.
- All patients achieved engraftment without clinically important GVHD.
- Co‐transplantation of MSCs may prevent GVHD without inhibition of engraftment.

**Lessons learned**

- Mesenchymal stem cells (MSCs) support hematopoiesis in the bone marrow and have immunomodulatory properties.
- MSCs have potencies to enhance engraftment and to prevent graft‐versus‐host disease (GVHD) after allogeneic hematopoietic cell transplantation.
- This study shows the safety of intra‐bone marrow co‐transplantation of MSCs in cord blood transplantation.
- All patients achieved engraftment without clinically important GVHD.
- Co‐transplantation of MSCs may prevent GVHD without inhibition of engraftment.

**Significance statement**

Mesenchymal stem cells (MSCs), which were derived from the bone marrow of third‐party donors, were injected into the bone marrow of the recipient 4 hours before cord blood transplantation. This study showed the safety of cord blood transplantation combined with intra‐bone marrow injection of MSCs and also suggested that cotransplantation of MSCs may prevent graft‐vs‐host disease without inhibition of engraftment. This strategy may be applicable not only to cord blood transplantation but also to bone marrow transplantation or peripheral blood stem cell transplantation, leading to the prevention of severe graft‐vs‐host disease, especially in human leukocyte antigen‐mismatched settings, and reduction of the burden imposed on hematopoietic stem cell donors by decreasing the required stem cell number.

## 1 INTRODUCTION

Cord blood transplantation (CBT) is a curative treatment for various hematologic disorders. Previous studies have reported comparable outcomes of CBT and human leukocyte antigen (HLA)‐matched unrelated donor transplantation in adult patients.^1^, ^2^ The incidence of chronic graft‐vs‐host disease (GVHD) is reported to be lower in CBT than in bone marrow transplant (BMT) and peripheral‐blood stem‐cell transplant (PBSCT); however, the incidence of acute GVHD in CBT is comparable with the other transplants.^1^, ^3^, ^4^, ^5^ In addition to a relatively high incidence of acute GVHD, delayed hematologic recovery and a higher rate of graft failure after CBT lead to an increased risk of transplant‐related mortality in the early period after transplantation.^3^, ^6^ Several strategies, including double‐unit CBT,^7^ ex vivo expansion of cord blood (CB)‐derived CD34+ cells,^8^, ^9^, ^10^, ^11^, ^12^ and intra‐bone marrow (BM) transplantation of CB cells,^13^, ^14^ have been explored in an effort to overcome these obstacles. In addition to these approaches, cotransplantation of CB and mesenchymal stem cells (MSCs) has been reported.^15^, ^16^, ^17^, ^18^

MSCs are a heterogeneous population of stromal stem cells that can be isolated from many tissues, such as BM, adipose tissue, CB, and placenta. MSCs have the capacity for self‐renewal and can differentiate into mesodermal lineage cells.^19^ In the BM, MSCs differentiate into BM stroma cells, osteocytes, osteoblasts, and endothelial cells. These cells contribute to the formation of the BM microenvironment, known as the hematopoietic stem cell (HSC) niche, and they support hematopoiesis.^20^, ^21^ Besides this hematopoietic support capacity, MSCs can modulate immune responses by a cell‐cell contact mechanism between MSCs and their target cells and by producing several soluble immunosuppressive factors.^19^, ^22^ These immunomodulatory effects of MSCs have already been clinically applied in the treatment of GVHD after allogeneic hematopoietic cell transplantation (HCT).^23^, ^24^, ^25^, ^26^, ^27^ MSCs express a low level of HLA class I and are negative for HLA class II and costimulatory molecules such as CD80, CD86, and CD40, and, therefore, they are able to evade allogeneic rejection.^28^, ^29^ Furthermore, culture expansion of MSCs is relatively easy, and they can be stored by cryopreservation. Therefore, ex vivo expanded and cryopreserved MSCs derived from a third‐party donor can be used for clinical treatment without considering HLA matching between patient and donor. Because of these properties, MSCs have been explored for enhancing engraftment and preventing GVHD after allogeneic HCT.

In previous clinical studies, the feasibility and safety of CBT with intravenous cotransplantation of MSCs were observed in pediatric patients.^15^, ^16^, ^17^, ^18^ However, to date, the cotransplantation of MSCs and CB cells has yet to be evaluated in adult patients, who have a greater risk of graft failure because of a lower CB cell dose per patient body weight. Concern regarding the route of MSC administration remains an issue because several animal model experiments have demonstrated that MSCs infused intravenously were trapped in lung,^30^, ^31^ and direct intra‐BM injection of MSCs could enhance the engraftment of transplanted CB cells more than intravenous injection.^32^ Additionally, intra‐BM injection of MSCs has been reported to be safe in previous clinical studies.^33^, ^34^

Based on these properties of MSCs and experimental and clinical findings, to develop a new strategy not only to enhance engraftment but also to prevent GVHD after CBT, a phase I trial of CBT combined with intra‐BM injection of ex vivo expanded MSCs (MSC‐CBT) was designed.^35^ The aim was to assess the safety of this treatment in adult patients with hematologic disorders.

## 2 MATERIALS AND METHODS

The study protocol has been described in detail previously.^35^ This study was registered with the University Hospital Medical Information Network Clinical Trials Registry (number 000024291).

### 2.1 Study design and participants

This was a single arm, nonrandomized, open‐label, single‐center, phase I trial at Nagoya University Hospital. The target sample size was five patients. Eligible patients were aged 20 years or older; had a hematologic disorder with an indication for CBT; did not have malignant cells accounting for 70% or more of all nucleated cells in the BM; had an Eastern Cooperative Oncology Group performance status of 0‐2; had adequate organ function as defined by an ejection fraction of 40% or greater, forced vital capacity of 50% or greater, forced expiratory volume in 1 second of 60% or greater, aspartate aminotransferase and alanine aminotransferase concentrations less than 5 times the upper limit of normal, and serum creatinine less than 3 times the upper limit of normal; and they had available CB units with serological HLA‐A, B, and DR ≥4/6 matched and with a total nucleated cell (TNC) dose of 1.5  ×  10^7^ cells per kg or higher. Additionally, patients had to have at least one potential MSC donor aged 20‐74 years from a spouse or relative within the fourth degree of relationship. The inclusion and exclusion criteria of the MSC donors are listed in the study protocol in detail.^35^

Patients were excluded if they had history of allogeneic HCT in the 1 year prior to enrollment, exposure to gemtuzumab ozogamicin in the 6 months prior to enrollment, and allergy to the drugs used for transplant preconditioning or GVHD prophylaxis. Exclusion criteria also included positive for HIV antibody, pregnancy or lactation, and uncontrolled psychiatric disorder or infection.

The Japanese Ministry of Health, Labour, and Welfare confirmed that this study complied with the Act on the Safety of Regenerative Medicine (number PA8160004, the latest edition version 5.1 22/Nov/2016). The study was conducted in accordance with the principles outlined in the Declaration of Helsinki, and all patients and donors provided written, informed consent before registration. Patients and donors were registered in this study after independent review by the data center in the Department of Hematology and Oncology, Nagoya University Graduate School of Medicine.

### 2.2 Preparation of MSCs

Human platelet lysates were prepared from single‐donor platelet concentrate provided by the Japan Red Cross Blood Center by the Application for the use of blood donated in Japan based on the “Guidelines on the use of donated blood in R&D, etc.” Platelet concentrate was frozen at −30°C and thawed twice and then stored at −30°C. The frozen platelet concentrate was thawed at 4°C and centrifuged to obtain supernatant as platelet lysate.

BM was harvested from the posterior iliac crest of the MSC donor with local anesthesia by the standard procedure of BM aspiration. The target MSC dose was 0.5 × 10^6^ cells per kilogram of patient weight. The volume of BM aspirate was determined according to patient weight; if the patient weight was <35, 35‐50, or ≥50 kg, the volume of BM aspirate was 10, 15, or 20 mL, respectively. Mononuclear cells were isolated by centrifugation of BM using Ficoll‐Paque PREMIUM (GE Healthcare Japan, Tokyo, Japan). The separated mononuclear cells were seeded in T‐25 cell culture flasks at 1.0‐2.0 × 10^7^ cells per flask in Dulbecco's modified Eagle's medium (Thermo Fisher Scientific, Waltham, Massachusetts) containing 5% human platelet lysate and 2 IU/mL heparin (Mochida Pharmaceuticals, Tokyo, Japan) (referred to as culture medium) and cultured at 37°C in a humidified incubator containing 5% CO_2_. After culturing for 3 or 4 days, nonadherent cells were removed, and the adherent cells were further cultured. Cells were harvested at subconfluent using TrypLE Select (Invitrogen, Carlsbad, California). Cells at passage 1 were seeded in the same number of T‐75 culture flasks from T‐25. Cells at passage 2 were seeded in the same number of T‐225 culture flasks from T‐75. Cells at passage 3 or 4 were seeded in a 3‐fold number of T‐225 flasks. MSCs were harvested after passage 3 or 4 according to the cell count, sampled, and cryopreserved at −150°C in CP‐1 (Kyokuto Pharmaceutical, Tokyo, Japan) until the day of CBT.

Criteria for release of MSCs for clinical use were as follows: viability ≥70%, viable cell count ≥0.2 × 10^6^ cells per kilogram of patient weight, absence of contamination by pathogens (as documented by a sterility test, endotoxin test, β‐D‐glucan assay, mycoplasma polymerase chain reaction (PCR) test, and viral PCR tests for hepatitis B and C viruses, HIV type 1, parvovirus B19, herpes simplex virus, varicella‐zoster virus, human herpesvirus‐6, cytomegalovirus, Epstein‐Barr virus), and immune phenotype characterized by the expression of CD73, CD90, and CD105 surface molecules (≥90%) and the absence of CD14, CD19, CD34, CD45, and HLA‐DR expressions (≤10%).

### 2.3 Conditioning regimen and GVHD prophylaxis

The conditioning regimen was not defined in this study. in vivo purging of T cells using treatments such as anti‐thymocyte globulin was prohibited. GVHD prophylaxis consisted of the combination of tacrolimus and short‐term methotrexate.

### 2.4 Cotransplantation of MSCs and CB cells

On the day of CBT, MSCs were thawed, washed, and resuspended in 2‐10 mL of a saline solution. Premedication with hydrocortisone 100 mg and chlorpheniramine 10 mg was administered approximately 30 minutes before injection of MSCs. After local anesthesia, a standard BM aspiration needle was inserted into the iliac bone on one side. To ensure that the needle was securely inserted into the BM cavity, aspiration of <0.5 mL BM was done. Then, approximately 5 mL of MSC suspension were injected slowly. This procedure was repeated on the iliac bone on the contralateral side. Four hours after MSC injection, CB was infused intravenously with the standard procedure. Granulocyte colony‐stimulating factor (G‐CSF) was administered from 7 days after transplant to neutrophil engraftment.

### 2.5 Follow‐up and assessment

Adverse events were graded by Common Terminology Criteria for Adverse Events version 4.0. Safety was assessed by monitoring and recording of all adverse events and serious adverse events. The study was monitored by an independent data and safety monitoring committee, and serious adverse events were reviewed and judged to determine whether an adverse event was attributable to treatment. Periodic monitoring was done according to the Japanese clinical trial guideline at least annually. The study stopping rules included graft failure, transplant‐related mortality before engraftment, or grade 4‐5 adverse event in three patients.

Patients had routine clinical assessments and laboratory investigations such as blood cell counts, tacrolimus levels, and cytomegalovirus antigenemia. Patients were planned to be followed up for at least 1 year after transplant or less if they satisfied one of the discontinuation criteria. Chimerism analyses were done at the National Hospital Organization Nagoya Medical Center using short tandem repeats by PCR assay in peripheral CD3+ T cells on days 14, 28, and 56 after transplantation as previously described.^36^ BM mononuclear cells (BM‐MNCs) and BM‐derived MSCs were also assessed for chimerism status including MSC donor chimerism on days 14, 28, 56, and 84 after transplantation. The presence of ectopic tissue formation was assessed by evaluating computed tomography scans taken 1 year after transplantation.

To evaluate immune reconstitution, lymphocyte subsets were measured at days 28, 42, 56, and 84 after transplantation by immunophenotyping of peripheral blood. Peripheral blood mononuclear cells (PBMCs) were stained with the following fluorochrome‐conjugated monoclonal antibodies: anti‐human CD3, CD4, CD8, CD16, CD19, CD25, CD27, CD56, and CD127 (BD Biosciences, San Jose, California). To stimulate interferon (IFN)‐γ, interleukin (IL)‐4, and IL‐17 production, the PBMCs were stimulated for 5 hours with 50 ng/mL phorbol myristate acetate and 1000 ng/mL ionomycin in the presence of brefeldin A (Golgiplug, BD Biosciences). Subsequently, the cells were fixed and permeabilized with Cytofix/Cytoperm Solution and Perm/Wash Buffer (BD Biosciences). After fixation, the cells were stained with IFN‐γ, IL‐4, IL‐17, and CD4 antibodies. Data acquisition and analyses were performed with a FACSAria (BD Biosciences) instrument.

To evaluate cytokine and chemokine kinetics, serum concentrations of IL‐1α, IL‐1β, IL‐2, IL‐4, IL‐5, IL‐6, IL‐10, IL‐12, IL‐13, IL‐17, IL‐21, IFN‐γ, tumor necrosis factor, IP‐10/CXCL10, and MCP‐1/CCL2 through pre‐ and post‐transplant (days 0, 1, 7, 14, 21, and 28) were measured using a Cytometric Bead Array Flex Set System (BD Biosciences), according to the manufacturer's instructions. On day 0, serum was obtained twice, before the infusions of MSCs and of CB cells. Samples were run on FACSCanto II (BD Biosciences), and data were analyzed using FCAP Array software (BD Biosciences). The changes in cytokine and chemokine levels from day 0 were evaluated.

### 2.6 Outcomes

The primary endpoint of this study was toxicity related to intra‐BM injection of MSCs within 14 days after transplantation, which was defined as adverse events that could not be explained by other complications, such as regimen‐related toxicity or infection, that generally occur after transplantation. Secondary endpoints included the rate of engraftment, the time to hematopoietic recoveries, the incidences and severities of acute and chronic GVHD, the incidences of regimen‐related toxicities and infection, and the probabilities of nonrelapse mortality (NRM), relapse, disease‐free survival, and overall survival at 1 year after transplantation.

### 2.7 Control comparison

The study patients were compared with a control group of six patients who received CBT without MSCs during the same time period, between May 2017 and May 2018, in Nagoya University Hospital with respect to hematopoietic recoveries, clinical outcomes, lymphocyte subsets, and cytokine/chemokine kinetics. The control patients did not join this study because of patient decisions (n = 1), not enough time to prepare MSCs (n = 1), the difficulty of BM aspiration due to BM fibrosis (n = 1), not meeting the criteria for release of MSCs (n = 1), or the close of registration for this study (n = 2).

### 2.8 Definitions and statistical analysis

Engraftment was defined as neutrophil recovery to greater than 0.5 × 10^9^/L for 3 consecutive days. The time to neutrophil engraftment was defined as the first day of achieving an absolute neutrophil count greater than 0.5 × 10^9^/L for 3 consecutive days. The times to platelet and reticulocyte recoveries were defined as the first days of achieving a platelet count greater than 20 × 10^9^/L or 50 × 10^9^/L and a reticulocyte count greater than 1% for 3 consecutive days without transfusions. Primary graft failure was defined as lack of neutrophil engraftment in patients surviving at least 60 days, and secondary graft failure was defined as neutrophil engraftment followed by a decline in the neutrophil count to less than 0.5 × 10^9^/L for 3 consecutive days. Acute GVHD was diagnosed and graded according to the consensus criteria.^37^ Chronic GVHD was evaluated according to the traditional Seattle criteria^38^ and the National Institutes of Health criteria for diagnosis and severity of chronic GVHD.^39^ Relapse was defined as recurrence of disease after transplantation. NRM was defined as death without disease relapse.

Mann‐Whitney tests and Fisher's exact tests were used to compare baseline characteristics and outcomes between the MSC‐CBT group and the control group. Mann‐Whitney tests were also used for the comparisons of lymphocyte subsets and cytokine/chemokine kinetics between the two groups. The probabilities of hematopoietic recoveries and GVHD were estimated on the basis of cumulative incidence curves and compared using Gray's test or the Fine and Gray competing risk regression model. Regarding hematopoietic recoveries, death and relapse were the competing events; for GVHD, death without GVHD and relapse were the competing events. All *P* values were two‐sided, and values of *P* < .05 were considered significant. Statistical analyses were performed with Stata software version 15.1 (StataCorp LP, College Station, Texas), GraphPad Prism software version 6.03 (GraphPad software, San Diego, California), and EZR software version 1.33.^40^

## 3 RESULTS

### 3.1 Characteristics of patients, grafts, and MSC donors

Between February 2017 and June 2018, six patients were enrolled in this study, but one patient did not receive protocol treatment because the MSC product did not meet release criteria because of insufficient cell counts (data of MSC expansion are summarized in Table 1). The characteristics and outcomes of five patients who received MSC‐CBT are summarized in Table 2. The median age was 47 years (range, 24‐70 years), and four patients (80%) were male. Two patients (40%) had acute myeloid leukemia in first or second complete remission, two (40%) had myelodysplastic syndrome with excess blasts‐2, and one (20%) had extranodal natural killer/T‐cell lymphoma in first remission. CB was serological HLA‐A, B, and DR 5/6 and 4/6 matched in both the graft‐vs‐host and the host‐vs‐graft direction in one case and four cases, respectively. The median number of cryopreserved TNCs and CD34+ cells in a CB unit was 2.56 × 10^7^/kg (range, 1.85‐4.35 × 10^7^/kg) and 0.99 × 10^5^/kg (range, 0.74‐1.18 × 10^5^/kg), respectively. No patients had donor‐specific HLA antibodies. All patients received a myeloablative conditioning regimen. MSC donors were HLA‐haploidentical in four cases (patient's son in three cases and mother in one case), and HLA‐mismatched in one case (patient's wife). The median number of infused MSCs was 1.35 × 10^6^/kg (range, 0.28‐1.79 × 10^6^/kg).

**TABLE 1:** Data of MSC expansion

Do no.	Age, y	Sex	Donor relation	BM volume, mL	BM‐MNCs, ×10^7^	Culture time, days	Passage number	MSCs, ×10^7^	MSCs, ×10^6^/kg	QC
1	25	F	Wife	20	8.7	29	3	7.8	1.43	Passed
2	28	M	Son	20	11.0	28	3	9.6	1.35	Passed
3	70	F	Mother	20	6.8	32	3	2.6	0.28	Passed
4	20	M	Son	15	9.8	29	3	9.0	1.79	Passed
5	58	M	Husband	15	4.8	35	3	0.4	0.08	Growth failure
6	42	M	Son	20	2.2	33	3	3.0	0.47	Passed

Abbreviations: BM, bone marrow; BM‐MNC, BM mononuclear cell; Do no., donor number; F, female; M, male; MSC, mesenchymal stem cell; QC, quality control.

**TABLE 2:** Characteristics of patients and outcomes of cord blood transplantation combined with intra‐bone marrow injection of MSCs

Patient no.	1	2	3	4	5
Patient and transplant					
Age, years	24	51	45	47	70
Diagnosis	MDS	AML	ENKTL	AML	MDS
Disease status	RAEB2	CR2	CR1	CR1	RAEB2
HCT‐CI	0	1	0	1	0
Sex, patient/donor	M/F	M/F	M/M	F/F	M/M
ABO, patient/donor	A/A	A/AB	A/B	A/B	B/B
DSA	Neg	Neg	Neg	Neg	Neg
Conditioning	CA + CY + TBI	Flu+Mel+ivBU	Flu+Mel+ivBU	CA + CY + TBI	Flu+Mel+ivBU
GVHD prophylaxis	TAC + sMTX	TAC + sMTX	TAC + sMTX	TAC + sMTX	TAC + sMTX
Cord blood					
TNCs, × 10^7^/kg	2.56	2.37	1.85	4.35	3.22
CD34 + cells, × 10^5^/kg	1.18	0.99	0.66	0.74	1.06
HLA matching					
GVH direction	4/6	4/6	5/6	4/6	4/6
HVG direction	4/6	4/6	5/6	4/6	4/6
MSCs					
Donor relation	Wife	Son	Mother	Son	Son
Donor age, years	25	28	70	20	42
MSCs, × 10^6^/kg	1.43	1.35	0.28	1.79	0.47
HLA matching					
GVH direction	2/6	3/6	4/6	3/6	3/6
HVG direction	3/6	3/6	3/6	3/6	4/6
Outcomes					
Time to recovery, days					
Neutrophils ≥0.5 × 10^9^/L	20	17	27	25	21
Reticulocytes ≥1%	29	35	36	39	35
Platelets ≥20 × 10^9^/L	29	37	48	44	38
Platelets ≥50 × 10^9^/L	52	46	57	53	42
Acute GVHD, grade	No	No	No	I	No
Chronic GVHD	No	No	No	No	No
Relapse	No	No	No	No	No
Status at day 365	Alive	Alive	Alive	Alive	Alive

Abbreviations: ABO, ABO blood type; AML, acute myeloid leukemia; CA + CY + TBI, cytarabine (8 g/m^2^) + cyclophosphamide (120 mg/kg) + total body irradiation 12 Gy; CR1, first complete remission; CR2, second complete remission; DSA, donor HLA‐specific antigen; ENKTL, extranodal natural killer/T‐cell lymphoma; F, female; Flu+Mel+ivBU, fludarabine (180 mg/m^2^) + melphalan (80 mg/m^2^) + intravenous busulfan (12.8 mg/kg); GVH, graft‐vs‐host; GVHD, graft‐vs‐host disease; HCT‐CI, hematopoietic cell transplantation‐specific comorbidity index; HLA, human leukocyte antigen; HVG, host‐vs‐graft; M, male; MDS, myelodysplastic syndrome; MSC, mesenchymal stem cell; Neg, negative; RAEB2, refractory anemia with excess blasts‐2; sMTX, short‐term methotrexate; TAC, tacrolimus; TNC, total nuclear cell.

### 3.2 Adverse events

No adverse events related to intra‐BM injection of MSCs, including swelling and prolonged pain at the injection site, embolism, and osteomyelitis, within 14 days after transplantation (primary endpoint) were observed. Regimen‐related toxicities within 28 days after transplantation are summarized in Table 3. Grade 3 oral mucositis, gastrointestinal toxicities, including nausea/vomiting and diarrhea, and infectious complications, all of which were febrile neutropenia before engraftment, were the main toxicities. No patient developed sinusoidal obstructive syndrome. There were two severe adverse events: one hypokalemia and one progressive multifocal leukoencephalopathy (PML) in two individuals. One patient (#5) developed grade 4 hypokalemia, which was believed to be associated with administration of diuretics and an antifungal drug and recovered with potassium supplementation. One patient (#3) developed PML 325 days after transplantation, which was confirmed by the detection of John Cunningham virus DNA in the cerebrospinal fluid by polymerase chain reaction; this patient died 428 days after transplantation. The association between intra‐BM injection of MSCs and development of PML was excluded because John Cunningham virus DNA was detected neither in the infused MSCs nor in its culture supernatant. No other serious infections, such as pulmonary infection, sepsis, and cytomegalovirus disease, were observed. No ectopic tissue formation was present 1 year after transplantation.

**TABLE 3:** Regimen‐related toxicities within 28 days after transplantation

Toxicity	Grade, n (%)			
	1	2	3	4
Oral mucositis	0 (0)	0 (0)	5 (100)	0 (0)
Gastrointestinal	2 (40)	0 (0)	3 (60)	0 (0)
Hepatic	2 (40)	0 (0)	2 (40)	0 (0)
Cardiac	0 (0)	0 (0)	0 (0)	0 (0)
Arrhythmia	0 (0)	0 (0)	0 (0)	0 (0)
Pulmonary	0 (0)	0 (0)	0 (0)	0 (0)
Renal/urinary	0 (0)	0 (0)	1 (20)	0 (0)
Skin	0 (0)	0 (0)	0 (0)	0 (0)
Neurological	0 (0)	0 (0)	0 (0)	0 (0)
Bleeding	0 (0)	0 (0)	0 (0)	0 (0)
Hypotension	0 (0)	0 (0)	0 (0)	0 (0)
Hypoxia	0 (0)	0 (0)	0 (0)	0 (0)
Arrhythmia	0 (0)	0 (0)	0 (0)	0 (0)
Thrombotic thrombocytopenic purpura/hemolytic uremic syndrome	0 (0)	0 (0)	0 (0)	0 (0)
Infection	0 (0)	0 (0)	0 (0)	0 (0)

### 3.3 Hematopoietic recovery

**FIGURE 1:**
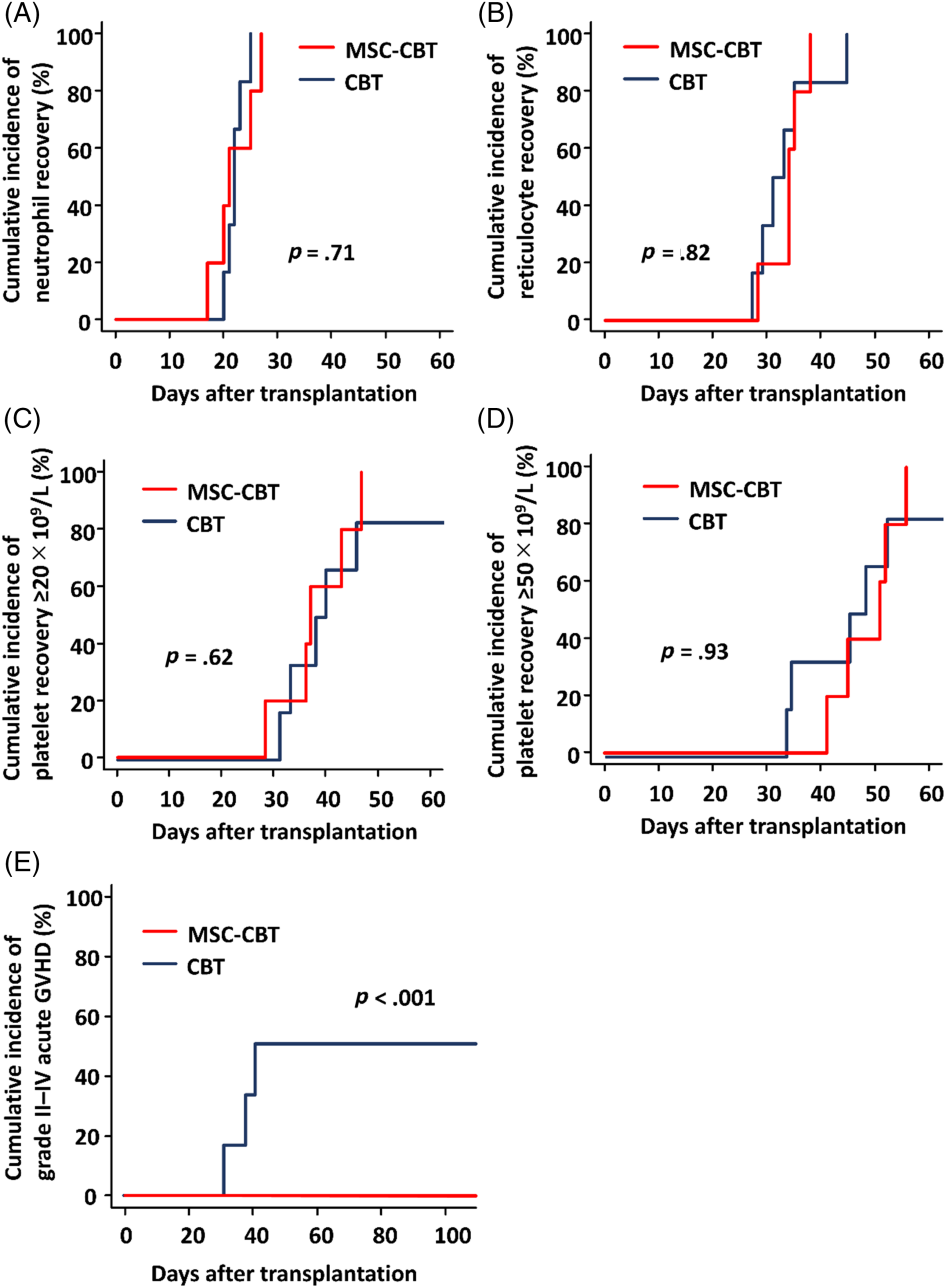
Hematopoietic recoveries and acute GVHD after MSC‐CBT and CBT alone. The cumulative incidences of A, neutrophil recovery ≥0.5 × 10^9^/L, B, reticulocyte recovery ≥1%, C, platelet recovery ≥20 × 10^9^/L, D, platelet recovery ≥50 × 10^9^/L, and E, grade II‐IV acute GVHD after transplantation are shown. CBT, cord blood transplantation; GVHD, graft‐vs‐host disease; MSC‐CBT, cord blood transplantation combined with intra‐bone marrow injection of mesenchymal stem cells

All patients achieved neutrophils ≥0.5 × 10^9^/L, reticulocytes ≥1%, platelets ≥20 × 10^9^/L, and platelets ≥50 × 10^9^/L, with median times to recovery of 21 (range, 17‐27), 35 (range, 29‐39), 38 (range, 29‐48), and 52 (range, 42‐57) days after transplantation, respectively (Table 2; Figure 1A‐D). No patient developed engraftment syndrome. All patients achieved complete (>95%) donor T‐cell chimerism at days 14, 28, and 56 after transplantation (Table S1).

### 3.4 MSC engraftment

BM aspirations were performed in all patients around days 28, 56, and 84 after transplantation. Chimerism of BM‐MNCs at any time point was complete CB donor type in all patients. Chimerism of MSCs expanded from BM‐MNCs at any time point was complete recipient type in all patients. No evidence of MSC donor chimerism was detectable in BM‐MNCs and BM‐derived MSCs at any time point in all patients (Table S1).

### 3.5 GVHD and survival

Only one patient experienced transient grade I acute GVHD of the skin, which improved without systemic immunosuppressive treatment. No patient developed grade II‐IV acute GVHD (Figure 1E), and no patient developed chronic GVHD. All patients were alive without relapse at 1 year after transplantation.

### 3.6 Control comparison

The control group consisted of six adult patients who received CBT without MSC during the same time period at our institution (Table S2). The comparisons of patient characteristics and transplant outcomes between MSC‐CBT patients and controls are summarized in Table 4. There was no significant difference between patients who received MSC‐CBT and controls in terms of patient characteristics. One control patient died with relapse of leukemia without achievement of platelet recovery (Table S2). The cumulative incidences of neutrophil, reticulocyte, and platelet recoveries were similar in the two groups (Figure 1A‐D). For those who achieved hematopoietic recovery, the median time to neutrophil, reticulocyte, and platelet recoveries was not significantly different between the two groups (Table 4). Grade II‐IV acute GVHD developed in three controls (50%); however, there was no grade II‐IV acute GVHD in MSC‐CBT patients. The cumulative incidence of grade II‐IV acute GVHD was significantly lower in MSC‐CBT patients compared with controls (Figure 1E). Chronic GVHD did not develop in both groups. The incidence of relapse, transplant‐related mortality, and overall survival were not significantly different between the two groups.

**TABLE 4:** Comparison between patients receiving MSC‐CBT and CBT alone

Characteristic or outcome	MSC‐CBT (n = 5)	CBT (n = 6)	*P* value
Patient characteristics			
Patient age, median (range), y	47 (24‐70)	44 (35‐52)	.62
Patient sex, male, n (%)	4 (80)	2 (33)	.24
Diagnosis, n (%)			.35
AML	2 (40)	3 (50)	
ALL	0 (0)	2 (33)	
MDS	2 (40)	0 (0)	
ENKTL	1 (10)	0 (0)	
ET	0 (0)	1 (17)	
Disease risk, advanced, n (%)	3 (60)	3 (50)	1.00
HCT‐CI, ≥2, n (%)	0 (0)	2 (33)	.46
Conditioning, MAC, n (%)	5 (100)	6 (100)	NA
GVHD prophylaxis, TAC + sMTX, n (%)	5 (100)	6 (100)	NA
Sex mismatch, n (%)	2 (40)	4 (67)	.57
ABO mismatch, n (%)	3 (60)	3 (50)	1.00
HLA matching, GVH direction, n (%)			1.00
5/6	1 (20)	1 (17)	
4/6	4 (80)	5 (83)	
HLA matching, HVG direction, n (%)			.46
5/6	1 (20)	0 (0)	
4/6	4 (80)	6 (100)	
DSA, negative, n (%)	5 (100)	6 (100)	NA
TNCs, median (range), × 10^7^/kg	2.56 (1.85‐4.35)	2.90 (2.26‐3.79)	.83
CD34 + cells, median (range), × 10^5^/kg	0.99 (0.66‐1.18)	0.66 (0.45‐0.94)	.07
Outcomes			
Graft failure, n (%)	0 (0)	0 (0)	NA
Time to recovery, median days (range)			
Neutrophils ≥0.5 × 10^9^/L	21 (17‐27)	22 (20‐25)	.85
Reticulocytes ≥1%	35 (29‐39)	33 (28‐46)	.58
Platelets ≥20 × 10^9^/L	38 (29‐48)	39 (32‐47)	1.00
Platelets ≥50 × 10^9^/L	52 (42‐57)	46 (34‐53)	.29
Acute GVHD, grade II‐IV, n (%)	0 (0)	3 (50)	.24
Acute GVHD, grade III‐IV, n (%)	0 (0)	1 (17)	1.00
Chronic GVHD, n (%)	0 (0)	0 (0)	NA
Relapse, n (%)	0 (0)	2 (33)	.46
1‐year TRM, n (%)	0 (0)	0 (0)	NA
1‐year OS, n (%)	5 (100)	5 (83)	1.00

Abbreviations: ABO, ABO blood type; ALL, acute lymphoblastic leukemia; AML, acute myeloid leukemia; CBT, cord blood transplantation; DSA, donor HLA‐specific antigen; ENKTL, extranodal natural killer/T‐cell lymphoma; ET, essential thrombocythemia; GVH, graft‐vs‐host; GVHD, graft‐vs‐host disease; HCT‐CI, hematopoietic cell transplantation‐specific comorbidity index; HLA, human leukocyte antigen; HVG, host‐vs‐graft; MAC, myeloablative conditioning; MDS, myelodysplastic syndrome; MSC‐CBT, cord blood transplantation combined with intra‐bone marrow injection of mesenchymal stem cells; NA, not available; OS, overall survival; sMTX, short‐term methotrexate; TAC, tacrolimus; TNC, total nuclear cell, TRM, transplant‐related mortality.

### 3.7 Lymphocyte subset and cytokine/chemokine analysis

Flow cytometry analysis was performed to compare lymphocyte reconstitution after transplantation between MSC‐CBT patients and controls. There were no significant differences in lymphocyte subsets at 28, 42, 56, and 84 days after transplantation between the two groups (Figure S1 and Table S3). Cytometric bead array analysis was performed to detect changes in cytokine and chemokine production with the addition of intra‐BM injection of MSCs. There were tendencies to decreases in IFN‐γ, IL‐1α, IL‐2, IL‐4, and IL‐21 levels within 28 days after transplantation in MSC‐CBT patients compared with controls (Figure S2).

## 4 DISCUSSION

The present study showed the safety of CBT combined with intra‐BM injection of MSCs for adult patients with hematologic disorders. Although several previous clinical studies had shown the safety and feasibility of cotransplantation of MSCs,^15^, ^16^, ^17^, ^18^, ^41^, ^42^, ^43^, ^44^, ^45^, ^46^ MSCs were intravenously infused in all except one study,^33^ and the combination of cotransplantation of MSCs and CBT was reported only in pediatric patients.^15^, ^16^, ^17^, ^18^ To the best of our knowledge, this is the first clinical trial of CBT with cotransplantation of MSCs in a cohort of adult patients and also the first trial of CBT combined with intra‐BM injection of MSCs. In the present study, no patient had adverse events related to intra‐BM injection of MSCs, and all patients achieved sustained engraftment and were alive at least 1 year after transplantation without grade II‐IV acute GVHD and relapse. Although MSCs have immunosuppressive properties, which are thought to be associated with the risk of delayed immune reconstitution and attenuation of graft‐vs‐tumor effects, immune reconstitution after transplantation was not inhibited, and relapse was not observed in MSC‐CBT patients in the present study. Though GVHD treatment with MSCs has been reported to be a risk factor for pneumonia‐related mortality,^47^ no patient developed pulmonary infection and severe infectious complications, at least in the early stage after transplantation.

Four clinical studies of cotransplantation of MSCs in CBT for pediatric patients have been reported. One study, in which G‐CSF was used only for 23% of the patients, reported a rate of neutrophil engraftment of 85%, with a median time of 30 days after transplantation and no reduction of the risk of graft failure.^16^ On the other hand, in the other studies, all patients achieved neutrophil engraftment at a median of 11, 19, and 19 days after transplantation, whereas the times of neutrophil and platelet recoveries were not different compared with historical control groups.^15^, ^17^, ^18^ Because these study subjects were pediatric patients and the total cell dose of the CB graft was sufficient (median 3.1 to 5.7 × 10^7^/kg), the differences in hematopoietic recoveries between with and without cotransplantation of MSCs were thought to be minimal. In the present study, all five adult patients achieved both neutrophil and platelet recoveries with relatively lower cell doses of CB graft compared with pediatric patients, including a unit with TNCs <2.0 × 10^7^/kg. Although there was no difference in the time of hematopoietic recoveries, the present study indicated the potential of intra‐BM injection of MSCs to support sustained engraftment. Further study is required to determine whether intra‐BM injection of MSCs improves engraftment in CBT for adult patients.

In the present study, the engraftment of cotransplanted MSCs was analyzed by chimerism analysis using short tandem repeats with a detection limit of 0.1%. The chimerism of MSCs derived from recipient BM after transplant remained of recipient origin and the fraction of the MSC donor was not detected. In the BM, MSCs differentiate into BM stromal cells, osteocytes, osteoblasts, and endothelial cells, all of which constitute the BM microenvironment, known as the HSC niche, and they support hematopoiesis by controlling maintenance, self‐renewal, proliferation, differentiation, and mobilization of HSCs.^20^ In addition, it has been suggested that MSCs play a role in the enhancement of homing and engraftment of HSCs to the BM niche by secreting various growth factors, cytokines, chemokines, and chemokine ligands, despite feeble and transient engraftment of MSCs themselves into BM.^48^, ^49^, ^50^, ^51^ Recently, MSC‐derived extracellular vesicles have been reported to increase the expression of CXCR4 on CB HSCs, paralleled by augmented homing of HSCs to the BM niche.^52^ In mouse model experiments, it has been demonstrated that direct intra‐BM injection of MSCs could enhance the engraftment of transplanted CB cells more than intravenous injection.^32^ This report also showed that significantly higher engraftment of CB cells was observed in not only BM into which MSCs were injected, but also the contralateral side BM without direct injection of MSCs.^32^ These findings imply that enhancement of engraftment of transplanted HSCs is obtained more by direct intra‐BM injection of MSCs, whereas it is not dependent on the sustained engraftment of donor MSCs in recipient BM.

Several inflammatory cytokines (IFN‐γ, IL‐1α, IL‐2, IL‐4, and IL‐21), which play important roles in the pathogenesis of GVHD, were lower in MSC‐CBT patients than in controls in this study. However, the comparison of cytokine levels has a limitation in the present small‐sample study. The observed differences in cytokine levels after transplant could be not the reason for, but rather the result of the lack of acute GVHD. Although all MSC‐CBT and control patients received similar myeloablative conditioning regimens, the observation could also be explained by differences in the actual damage caused by conditioning regimen. The effect of cotransplantation of MSCs on cytokine production and its association with the development of GVHD should be analyzed by further studies with larger cohorts of patients and controls.

In adult patients undergoing CBT in Japan, the incidences of grade II‐IV and grade III‐IV acute GVHD were reported to be 13%‐41% and 8%‐12%, respectively.^2^, ^3^, ^6^ Although no development of grade II‐IV acute GVHD in MSC‐CBT patients suggests the potential that cotransplantation of MSCs may prevent GVHD, this should be confirmed by further studies, and a phase II trial to evaluate the efficacy of this strategy is now being planned.

## 5 CONCLUSION

CBT combined with intra‐BM injection of MSCs was found to be a safe and feasible therapeutic strategy. Furthermore, the present findings suggest the potential that intra‐BM injection of MSCs may prevent the development of GVHD, accompanied by a decrease in some inflammatory cytokines, with no inhibition of engraftment and immune reconstitution. This strategy might be applicable not only to CBT but also to BMT or PBSCT, leading to prevention of severe GVHD, especially in HLA‐mismatched settings and reduction of the burden imposed on HSC donors by decreasing the required stem cell number. Further clinical trials are needed to confirm the efficacy of intra‐BM injection of MSCs.

## CONFLICT OF INTEREST

T.M. received educational and investigational support from Chugai Pharmaceutical Co., Ltd. and Novo Nordisk Pharma Ltd.; honoraria from Shire/Takeda Pharmaceutical Co., Ltd., Bayer Pharmaceutical Co., Ltd., Bioverativ/Sanofi Co., Ltd., Chugai Pharmaceutical Co., Ltd., CSL Behring Co., Ltd., and Novo Nordisk Pharma Ltd.; serves on advisory boards for Baxalta/Shire/Takeda Pharmaceutical Co., Ltd., Bayer Pharmaceutical Co., Ltd., Novo Nordisk Pharma Ltd., Chugai Pharmaceutical Co., Ltd., and Pfizer Co., Ltd. H.K. received research funding from FUJIFILM Corporation, Kyowa Hakko Kirin Co., Ltd., Otsuka Pharmaceutical Co., Ltd., Perseus Proteomics Inc., Daiichi Sankyo Co., Ltd., AbbVie GK, Astellas Pharma Inc., Zenyaku Kogyo Co., Ltd., Nippon Shinyaku Co., Ltd., Eisai Co., Ltd., Chugai Pharmaceutical Co., Ltd., Takeda Pharmaceutical Co., Ltd., and Sumitomo Dainippon Pharma Co., Ltd.; honoraria from Bristol‐Myers Squibb, Ltd. and Astellas Pharma Inc.; served as the Consultant/Advisory role Astellas, Daiichi Sankyo, received honoraria from Astellas, and received research funding from Chugai, Kyowa Hakko Kirin, Zenyaku Kogyo, FUJIFILM, Daiichi Sankyo, Astellas, Otsuka, Nippon Shinyaku, Eisai, Pfizer, Takeda, Novartis, Sumitomo Dainippon, Sanofi, Celgene. These companies were not directly involved in any part of this study. The other authors indicated no potential conflicts of interest.

## author contributions

T.G.: conception and design, provision of study material or patients, collection and/or assembly of data, data analysis and interpretation, manuscript writing, final approval of manuscript; M.M.: conception and design, financial support, provision of study material or patients, collection and/or assembly of data, data analysis and interpretation, manuscript writing, final approval of manuscript; T.N.: conception and design, provision of study material or patients, collection and/or assembly of data, data analysis and interpretation, final approval of manuscript; S.T., Y.I., Y.U., and Y.A.: provision of study material or patients, collection and/or assembly of data, final approval of manuscript; S.K.: collection and/or assembly of data, data analysis and interpretation, final approval of manuscript; S.S.: conception and design, provision of study material or patients, collection and/or assembly of data, final approval of manuscript; K.K., A.H., S.N., N.N., and Y.T.: conception and design, administrative support, final approval of manuscript; Y.K. and T.M.: administrative support, final approval of manuscript; H.K.: conception and design, financial support, administrative support, final approval of manuscript.

## Supporting information


**Figure S1.** Supporting information.

**Figure S2.** Supporting information.

**Table S1.** Chimerism analysis.
**Table S2.** Characteristics and outcomes of control patients receiving cord blood transplantation alone.
**Table S3.** Lymphocyte subset analysis.

## Data Availability

The data that support the findings of this study are available on request from the corresponding author.
