# Supplementary figures and images for: Phase I clinical trial of intra‐bone marrow cotransplantation of mesenchymal stem cells in cord blood transplantation
Source: Stem Cells Transl Med. 2020 Dec 14;10(4):542–53. doi: 10.1002/sctm.20-0381 (PMC7980216; doi:10.1002/sctm.20-0381)

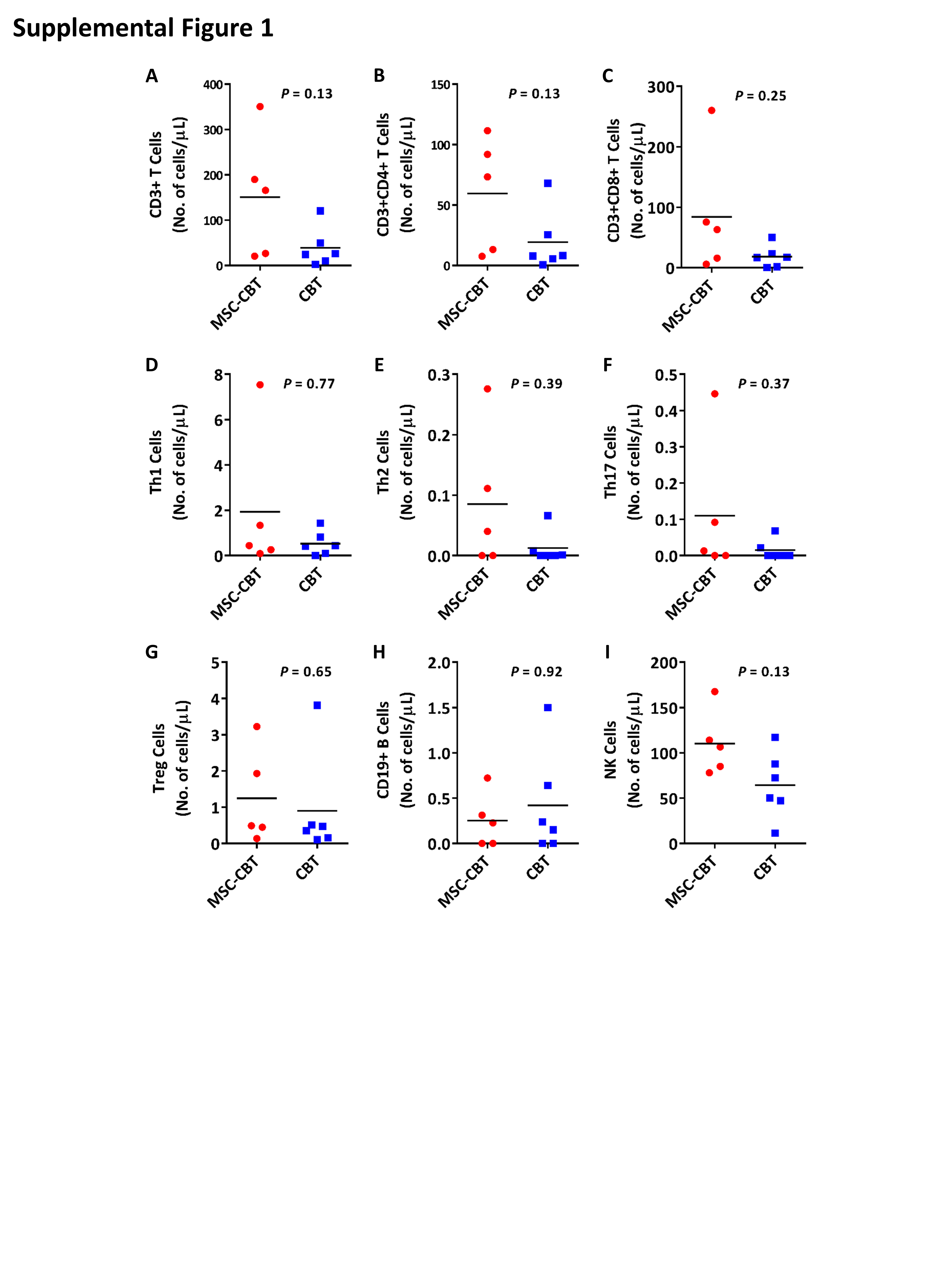

Supplement: Supplementary file 1 — Figure S1. Supporting information. [file SCT3-10-542-s002.TIF]

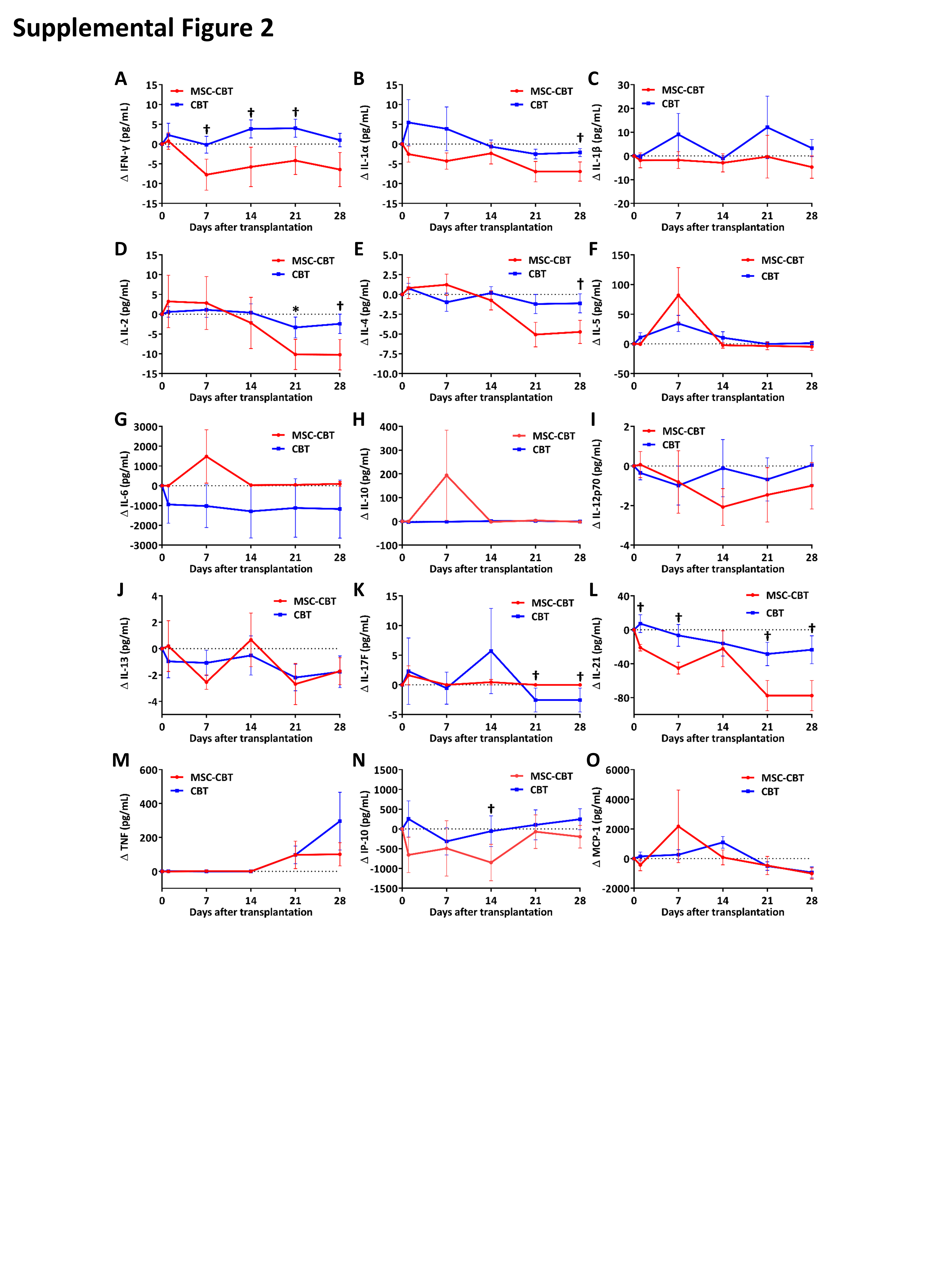

Supplement: Supplementary file 2 — Figure S2. Supporting information. [file SCT3-10-542-s003.TIF]
